# A Case of Diffuse Scleroderma Presenting as Hypertensive Encephalopathy Due to Scleroderma Renal Crisis

**DOI:** 10.7759/cureus.61732

**Published:** 2024-06-05

**Authors:** Sarang S Raut, Sourya Acharya, Sunil Kumar, Anil Wanjari, Nidhi A Bardiya

**Affiliations:** 1 General Medicine, Jawaharlal Nehru Medical College, Wardha, IND

**Keywords:** acute kidney injury, angiotensin-converting enzyme (ace) inhibitor, hypertensive encephalopathy, scleroderma renal crisis, diffuse scleroderma

## Abstract

This case report details a rare presentation of diffuse scleroderma, where a 38-year-old female developed hypertensive encephalopathy due to scleroderma renal crisis (SRC). SRC, characterized by sudden severe hypertension and renal failure, poses a life-threatening emergency. The patient's clinical features, including skin changes and abnormalities on nailfold capillaroscopy, facilitated the diagnosis of diffuse scleroderma. Comprehensive diagnostic investigations revealed multisystem involvement. Management involved angiotensin-converting enzyme inhibitors, hydroxychloroquine, and packed red cell transfusions, highlighting a holistic therapeutic approach. This case underscores the importance of recognizing diverse scleroderma manifestations in hypertensive emergencies for timely intervention and improved outcomes.

## Introduction

Systemic sclerosis (SSc), commonly known as scleroderma, is a rare autoimmune disease affecting primarily women of childbearing age. Characterized by progressive fibrosis of the skin and connective tissues, it can also involve internal organs such as the heart, lungs, kidneys, and intestines. The disease has three distinct phenotypes: limited scleroderma, diffuse scleroderma, and overlap syndrome. Limited scleroderma exhibits skin thickening mainly below the elbows and knees, with a higher risk of pulmonary arterial hypertension but less internal organ involvement. Diffuse scleroderma presents with widespread skin thickening, joint pain, and a greater propensity for internal organ fibrosis. Overlap syndrome manifests with features of scleroderma alongside other autoimmune diseases. While the etiology remains enigmatic, early diagnosis and comprehensive management involving medications, physical therapy, and lifestyle modifications are crucial for controlling symptoms and enhancing the quality of life for individuals living with this complex condition [1].

The renal manifestations of scleroderma can occur, and their presentation can range from mild proteinuria and hematuria to marked hypertension and progressive and profound renal failure called scleroderma renal crisis (SRC) [2]. SRC is characterized by sudden, severe high blood pressure and rapidly progressive renal failure. Administering angiotensin-converting enzyme (ACE) inhibitors early improved care and greatly reduced fatalities. However, SRC remains a medical emergency requiring swift action to safeguard kidney function and long-term survival [3]. On looking at the pathophysiology of SRC, the destructive process begins with subtle injury to the vessels' lining in the kidney, exploited by autoantibodies and inflammatory factors. This weakens the barrier, leading to dangerous fluid infiltration, edema, and an overactive coagulation cascade. Scar tissue formation, a characteristic of SSc, further constricts the arteries, aggravating the cycle of hypertension and renal damage. The relentless assault compromises kidney function, pushing patients toward renal failure. Swift treatment is crucial, involving medications to lower blood pressure and slow kidney damage. In severe cases, dialysis may be necessary. Understanding this complex process is vital for effective interventions and preventing the devastating impact of SRC [4].

Systemic scleroderma affects the central nervous system. However, the exact processes via which systemic scleroderma damages the nervous system at the pathogenetic level are still unknown. Specifically, T cells and dendritic cells are thought to have a significant impact because of their ability to produce cytokines, which ultimately cause vasculitis and damage to the dorsal root ganglia as a result of inflammatory infiltration. In addition, studies are being conducted to pinpoint particular antibodies that can trigger reactions against neural framework antigens. Cognitive deficits, aseptic meningitis, non-infectious seizures, persistent headaches, transverse myelitis, optic nerve inflammation, and diffuse encephalopathy are among the many symptoms that affect the central nervous system. Among these symptoms, cognitive deficits are one that people with systemic scleroderma frequently experience [5]. High blood pressure in SRC can lead to hypertensive encephalopathy due to central nervous system involvement. In such cases, lowering blood pressure is the aim of treating this medical emergency, and ACE inhibitors are beneficial and can cause a complete reversal of end-organ damage [6].

This case report presents a patient with diffuse SSc who embarks on a particularly treacherous turn in the scleroderma dance. The sudden rise in blood pressure, a consequence of SRC, leads to a perilous complication known as hypertensive encephalopathy. This life-threatening condition arises from the detrimental effects of uncontrolled hypertension on the central nervous system. In such cases, lowering blood pressure becomes the primary countermeasure, and ACE inhibitors prove invaluable tools, offering the potential for complete reversal of end-organ damage [7].

## Case presentation

A 38-year-old female was brought to the casualty by her relatives with chief complaints of confusion for the last six hours and being disoriented to time, place, and person within the same time period. Additionally, the patient had a history of dysphagia and recurrent regurgitation of food post-meals for the past four months and a history of recurrent oral gingival sores in the past year. The patient had no history of any comorbidities and was not on any medications.

**Figure 1:**
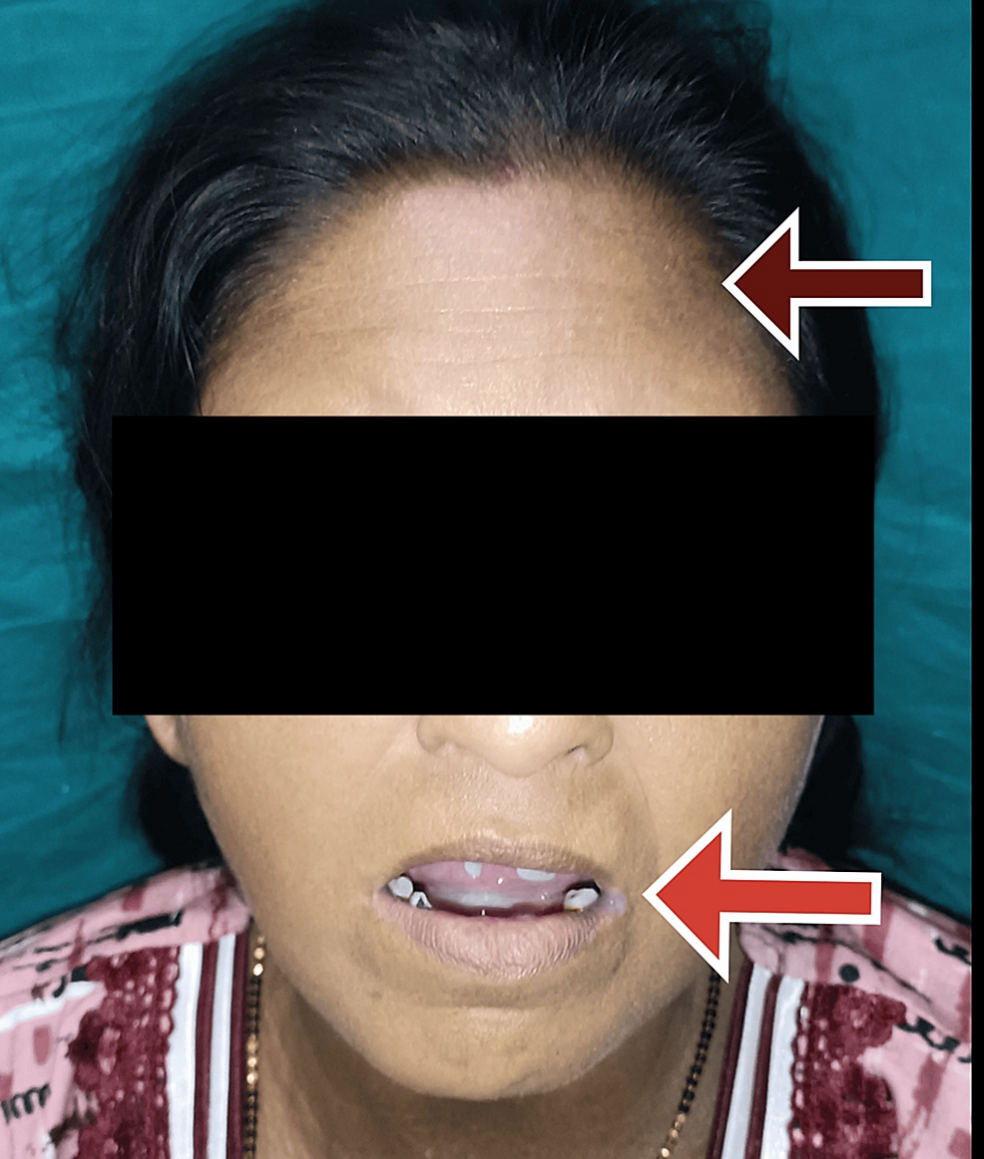
Image showing a pepper pot appearance on the forehead (brown arrow) and a limited mouth opening (red arrow)

**Figure 2:**
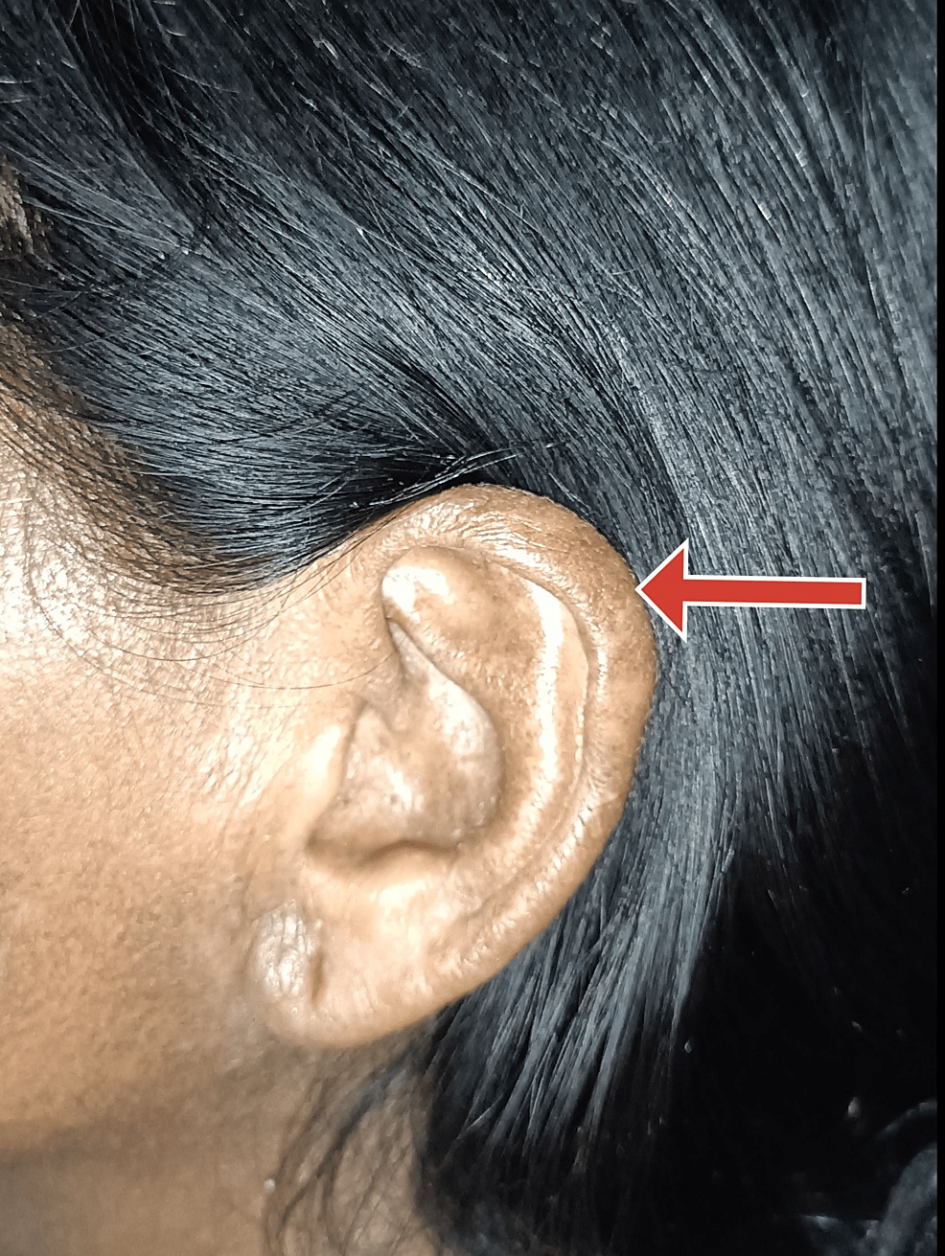
Image showing a pepper pot appearance of the ear (red arrow)

**Figure 3:**
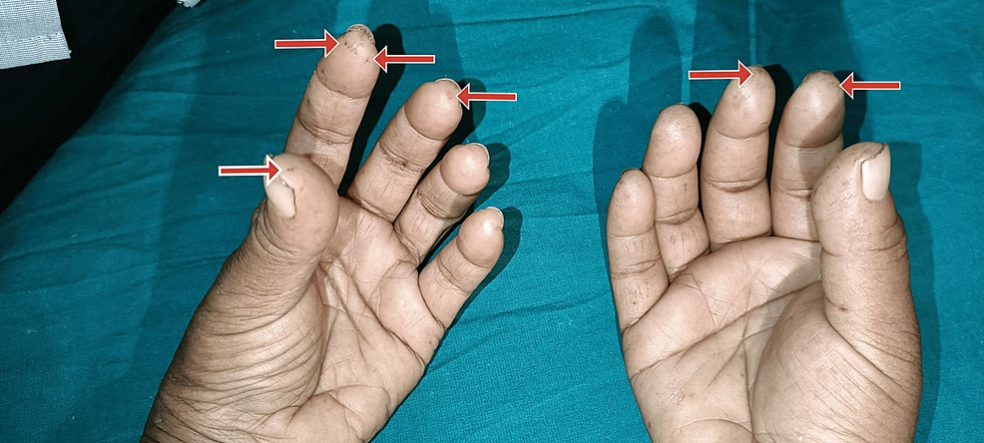
Image showing digital pits on both hands (red arrows)

**Figure 4:**
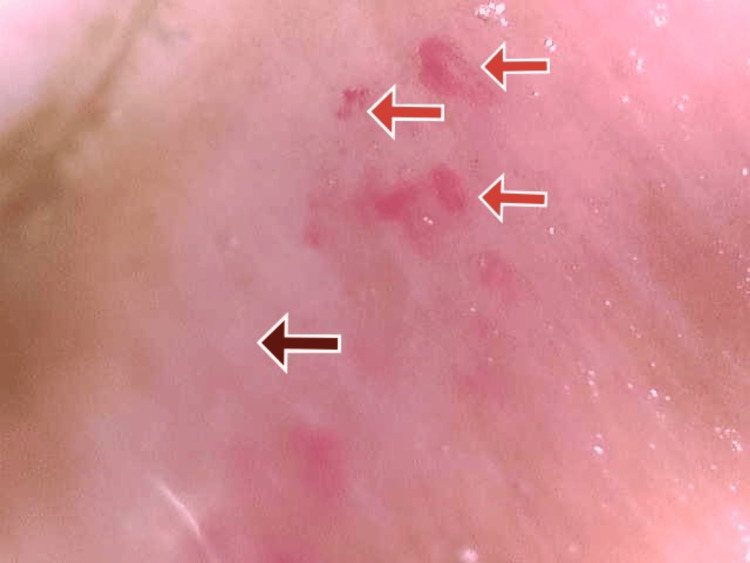
An image of nailfold capillaroscopy showing areas of giant capillaries (red arrows) and areas of capillary dropouts (brown arrow)

On general examination, the patient was irritable, pulse was 110 beats per minute, and blood pressure was 220/120 mmHg. The patient had no pallor, icterus, clubbing, cyanosis, pedal edema, or lymphadenopathy. The patient presented with darkened and hard skin in the neck and limb areas. The skin could not be pinched. The mouth opening of the patient was limited, as shown in Figure 1. There was a pepper pot appearance over the forehead and bilateral ears, as shown in Figures 1, 2. There were a few digital pits present in both hands, as shown in Figure 3. Figure 4 displays the presence of giant capillaries with capillary dropouts observed on nailfold capillaroscopy. Hence, a diagnosis of diffuse scleroderma was considered.

On a systemic physical examination, the cardiovascular system, respiratory system, and abdominal examination were normal. On the central nervous system examination, the patient was irritable and disoriented to time, place, and person. The tone was normal in all four limbs. Power could not be assessed as the patient was disoriented and irritable. Bilateral plantar reflexes were flexor, and all deep and superficial tendon reflexes were present. A diagnosis of hypertensive encephalopathy was considered. The patient's laboratory blood investigations upon admission to the hospital are given in Table 1, which were suggestive of acute kidney injury.

**Table 1:** Laboratory investigations of the patient

Lab parameters	Normal range	Observed values on day 1 of admission	Observed values on day 3 of admission	Observed values prior to discharge
Hemoglobin	13-17 g%	7.5 g%	8.9 g%	10.2 g%
Total leucocyte count	4000-11000 cell/mm^3^	6300 cell/mm^3^	8300 cells/mm^3^	6700 cells/mm^3^
Platelets	150000-400000 cell/mm^3^	1.92 lacs cell/mm^3^	1.86 lacs cells/mm^3^	1.94 lacs cells/mm^3^
Mean corpuscular volume	83-101 fL	74 fL	81 fL	88 fL
Serum creatinine	0.66-1.25 mg/dL	3.1 mg/dL	2.6 mg/dL	1.1 mg/dL
Serum urea	19-43 mg/dL	94 mg/dL	69 mg/dL	32 mg/dL
Serum sodium	137-145 mmol/L	138 mmol/L	139 mmol/L	140 mmol/L
Serum magnesium	1.6-2.3 mg/dL	1.9 mg/dL	2.0 mg/dL	2.0 mg/dL
Serum calcium	8.4-10.2 mg/dL	8.4 mg/dL	8.3 mg/dL	8.9 mg/dL
Serum potassium	3.5-5.1 mmol/L	4.1 mmol/L	4.2 mmol/L	4.5 mmol/L
Serum alkaline phosphatase	38-126 U/L	74 U/L	40 U/L	50 U/L
Serum aspartate transaminase	17-59 U/L	17 U/L	22 U/L	20 U/L
Serum alanine transaminase	<50 U/L	14 U/L	15 U/L	18 U/L
Serum albumin	3.5-5 g/dL	3.5 g/dL	3.6 g/dL	4.0 g/dL
Serum total bilirubin	0.2-1.3 mg/dL	0.5 mg/dL	0.2 mg/dL	0.5 mg/dL
Anti-Scl 70 IgG antibody	-	33 U/mL	not done	not done
International normalized ratio	<1.1	1	1	1.1

To confirm the diagnosis of diffuse scleroderma, an extractable nuclear antigen (ENA) profile of the patient was examined, which was suggestive of strong positivity for anti-scleroderma 70 IgG antibodies, as shown in Table 2. The evaluation of the ENA profile in Table 2 is given in Table 3. Hence, the diagnosis of SRC was considered as laboratory blood investigations were suggestive of acute kidney injury.

**Table 2:** The extractable nuclear antigen (ENA) profile of the patient Methodology: line immunoassay Bands are evaluated in relation to the cut-off band (index = 1)

Name of the antigen	Results	Index value
ds DNA (double-stranded DNA)	Negative	0.11
Nucleosome	Negative	0.19
Histones	Negative	0.15
SmD1 (SmD1-amino-acid 83-119 peptide)	Negative	0.11
PCNA (proliferating cell nuclear antigen)	Negative	0.19
PO (ribosomal P protein)	Negative	0.33
SS-A/Ro60	Negative	0.33
SS-A/Ro52	Negative	0.26
SS-B/La	Negative	0.67
CENP-B (centromere protein-B)	Negative	0.13
Scl70 (scleroderma-70)	Strongly positive	5
U1-snRNP (small nuclear ribonucleoprotein particle)	Negative	0.19
AMAM2 (anti-mitochondrial M2)	Negative	0.15
Jo-1 (myositis-specific)	Negative	0.11
PM-Scl (polymyositis-scleroderma)	Negative	0.15
Mi-2 (myositis-specific)	Negative	0.11
Ku (Ku antigen)	Negative	0.22
DFS-70 (dense fine speckled-70)	Negative	0.11

**Table 3:** The evaluation of Table 2 is based on the following rules

Evaluation	Symbol	Index
Negative	Without symbol	<0.2
Negative	-	≥0.2 and <0.8
Borderline	0	≥0.8 and <1.15
Weakly positive	+	≥1.15 and <2.5
Positive	++	≥2.5 and <4
Strongly positive	+++	≥4

The patient underwent a comprehensive diagnostic assessment, including various imaging studies. A 2D echocardiography was performed, revealing a well-maintained cardiac function with an ejection fraction of 60%, no evidence of regional wall motion abnormalities, and mild findings suggestive of grade 1 diastolic dysfunction. Additionally, the echocardiogram indicated mild tricuspid and mitral regurgitation, along with a right ventricular systolic pressure of 34 mmHg, indicative of mild pulmonary hypertension. Further investigations with abdominal and pelvic ultrasound unveiled the normal size of both kidneys with the left kidney being 9.1 cm in length and 4.1 cm in width and the right kidney being 8.8 cm in length and 3.6 cm in width with normal echotexture of both kidneys. A high-resolution computed tomography (CT) scan of the thorax was conducted to scrutinize the chest region and identify any lung pathology. The CT scan also revealed a sliding-type hiatal hernia, positioning the gastroesophageal junction 7 cm above the esophageal hiatus. Esophageal dilatation was observed from the level of D3 to D5 vertebral levels, with a maximum diameter measuring 1.2 cm.

In response to the symptoms of dysphagia, recurrent regurgitation of food, and the presence of a hiatal hernia, an upper gastrointestinal endoscopy was performed. The endoscopic examination confirmed reflux esophagitis of LA grade B and identified a Hill’s grade III hiatus hernia. This thorough assessment provided valuable insights into the structural abnormalities affecting the upper gastrointestinal tract. Concomitantly, a colonoscopy was conducted to evaluate the lower gastrointestinal tract as the patient was anemic. Fortunately, this examination yielded normal findings, contributing to a comprehensive understanding of the patient's gastrointestinal health.

For the treatment, the patient was started with intravenous enalaprilat to control blood with a dose of 1.25 mg intravenously every six hours, which is an ACE inhibitor. The serum creatinine of the patient was monitored as the patient was given ACE inhibitors although the patient has acute kidney injury. However, there was no observed increase in the patient’s serum creatinine levels (the laboratory blood investigations of the patient on the day of admission, on day 3, and at the time of discharge are given in Table 1). Additionally, as the patient was diagnosed with diffuse scleroderma, she was started with oral hydroxychloroquine at the dose of 200 mg twice a day. Two packed red cell transfusions were given to the patient because of severe iron deficiency anemia. Subsequently, the patient’s blood pressure returned to normal limits, and the results of renal function tests also fell within the normal range, as shown in Table 1. In addition, the patient received supportive management. She was discharged with tab enalapril 5 mg twice a day and tab hydroxychloroquine 200 mg twice a day for three months.

In summary, the patient presented with hypertensive encephalopathy, but the underlying etiology of this condition remained unknown. On careful evaluation, the patient was found to have clinical features of diffuse scleroderma, which was confirmed with laboratory investigations. The patient's renal function tests were deranged, leading to a diagnosis of SRC. The SRC was managed with intravenous enalaprilat.

## Discussion

This case report illuminates a critical instance of diffuse scleroderma presenting as a hypertensive emergency with encephalopathy, ultimately diagnosed as SRC. The complexity of SSc becomes evident as the patient's clinical picture unfolds, highlighting the importance of recognizing atypical presentations of this autoimmune disorder.

The patient's initial neurological symptoms of irrelevant talks and disorientation posed a diagnostic challenge, initially raising suspicion of essential hypertension presenting as hypertensive encephalopathy. However, a meticulous clinical examination revealed characteristic features of diffuse scleroderma, including skin changes, limited mouth opening, digital pits, and abnormalities on nailfold capillaroscopy. The recognition of these clinical hallmarks facilitated the accurate diagnosis of diffuse scleroderma [8].

The elevated blood pressure levels, reaching hypertensive urgency and ultimately causing hypertensive encephalopathy, were a consequence of SRC, emphasizing the need for swift intervention to prevent end-organ damage. The pathophysiological cascade involving vessel injury, inflammatory factors, and scar tissue formation elucidates the intricate mechanisms leading to hypertension and renal compromise in SRC, leading to acute kidney injury [9]. The prompt initiation of treatment with intravenous enalaprilat, which is an ACE inhibitor, was pivotal to mitigating the hypertensive crisis [10,11].

Diagnostic investigations, including 2D echocardiography, abdominal ultrasound, and a high-resolution CT scan, provided a comprehensive understanding of the systemic impact of diffuse scleroderma. Pulmonary hypertension, splenomegaly, and esophageal abnormalities reflected the multisystem involvement characteristic of this autoimmune disorder [12].

Initiating a treatment plan involving the use of an ACE inhibitor for blood pressure management, together with oral hydroxychloroquine (HCQ), has been found to be beneficial. HCQ has demonstrated a noteworthy decrease in serum levels of vascular cell adhesion molecule-1, E-selectin, endothelin-1, and NEMO videocapillaroscopy score in SSc. Consequently, a three-month course of HCQ leads to substantial enhancements in diffuse microvascular involvement. Severe iron deficiency anemia was addressed with two units of packed red cell transfusions. This demonstrates a holistic therapeutic approach targeting both the autoimmune nature of the disease and its complications [13].

## Conclusions

Diffuse scleroderma can be difficult to diagnose and treat, particularly when it manifests as unusual symptoms such as neurological manifestations. The hypertensive crisis in SRC can be efficiently managed with intravenous enalaprilat, but early detection is essential to avoid irreparable damage. Optimizing disease management requires a multifaceted strategy that includes hydroxychloroquine, ACE inhibitors, and treating consequences, including severe anemia. This emphasizes the value of continuing research and a multidisciplinary strategy to enhance patient outcomes.
